# Additive manufacturing process selection for automotive industry using Pythagorean fuzzy CRITIC EDAS

**DOI:** 10.1371/journal.pone.0282676

**Published:** 2023-03-09

**Authors:** Akin Menekse, Adnan Veysel Ertemel, Hatice Camgoz Akdag, Ali Gorener

**Affiliations:** 1 Istanbul Technical University, Istanbul, Turkey; 2 Faculty of Management, Istanbul Technical University, Istanbul, Turkey; 3 Faculty of Management, Istanbul Commerce University, Istanbul, Turkey; Libyan Academy, LIBYA

## Abstract

For many different types of businesses, additive manufacturing has great potential for new product and process development in many different types of businesses including automotive industry. On the other hand, there are a variety of additive manufacturing alternatives available today, each with its own unique characteristics, and selecting the most suitable one has become a necessity for relevant bodies. The evaluation of additive manufacturing alternatives can be viewed as an uncertain multi-criteria decision-making (MCDM) problem due to the potential number of criteria and candidates as well as the inherent subjectivity of various decision-experts engaging in the process. Pythagorean fuzzy sets are an extension of intuitionistic fuzzy sets that are effective in handling ambiguity and uncertainty in decision-making. This study offers an integrated fuzzy MCDM approach based on Pythagorean fuzzy sets for assessing additive manufacturing alternatives for the automotive industry. Objective significance levels of criteria are determined using the Criteria Importance Through Inter-criteria Correlation (CRITIC) technique, and additive manufacturing alternatives are prioritized using the Evaluation based on Distance from Average Solution (EDAS) method. A sensitivity analysis is performed to examine the variations against varying criterion and decision-maker weights. Moreover, a comparative analysis is conducted to validate the acquired findings.

## 1 Introduction

There are various alternatives for designing a product or conducting a particular manufacturing process for any given production application [1]. Methodical consideration must be given to the process selection for a given industrial task [2]. Decision-makers require a systematic and precise procedure for analyzing material and manufacturing alternatives [3]. Additive manufacturing (AM) provides tremendous promise for both product and process innovation across a broad spectrum of business sectors [4]. Because of its unique characteristics, AM has become an important methodology in modern production systems [5]. In contrast to subtractive manufacturing techniques, which involve cutting away material in the form of a block to create a finished product, “AM” describes a method of production in which material is added layer by layer, materials are joined to construct a part from a 3D model [6]. According to the American Society for Testing and Materials (ASTM) International, the different AM processes are divided into seven categories: Vat Photopolymerisation (VAT), Powder Bed Fusion (PBF), Material Extrusion (ME), Material Jetting (MJ), Binder Jetting (BJ), Sheet Lamination (SL), and Directed-Energy Deposition (DED) [7]. These processes also have alternatives inside themselves. All processes use an additive manufacturing technology to construct their parts, which allows for complex geometries to be made in a single process without the need for assembly [8].

AM’s layer-wise manufacturing method gives it unique advantages, such as more flexible manufacturing, more freedom in design, reduction of raw material waste and less cost and time spent on production [9, 10]. These experiences reveal the features of a disruptive innovation, which may radically alter existing business models but also create new ones [11]. AM is a solution that may give significant benefits, especially to the elimination of defects in products and the shortening of lead times by manufacturing at or near the required location [12].

Additionally, several industries including aerospace, automotive, engineering, medical, and biological systems have lately used various AM technologies to produce end-user tailored products [13]. In 2021, the global market for additive manufacturing was worth USD 13.84 billion, and it is expected to grow at a rate of 20.8% per year from 2022 to 2030. There were 2.2 million 3D printers shipped worldwide in 2021, and that number is projected to rise to 21.5 million by the year 2030 [14]. Despite these findings, industrial applications of additive manufacturing are still insufficient, hence study on the selection of AM processes relevant to the industry and material is necessary [15].

In the automotive industry, AM’s tool-free nature helps to simplify supply chains and provide more agile and sustainable opportunities [16]. Besides the main benefits of AM mentioned above, the automotive industry can reduce the weight of a vehicle by replacing heavy metal parts with AM fabricated composite materials that have stiffness and strength-to-weight ratios. Moreover, the adoption of AM may significantly boost productivity, decrease lead time, and lower the cost of manufacturing molds and dies [17]. Both internal and external factors pose a significant risk to the automotive industry’s supply chain. Abrupt discontinuity of a supplier, machine breakdown, quality problems, malfunction of digital technologies, and delivery chain delays are all examples of internal risks. Also included among the external risks are the possibilities of an accident, an import restriction, demand uncertainties, a natural disaster, a pandemic, a labor strike, a rise in customs duties, fuel price increase and other economic risks [18–21]. The automotive industry is one of the main industries for potential AM applications because of its complex and long supply chain [22].

The objective of the research is to analyze the suitability of additive manufacturing processes for the automotive industry, which has always played a significant role in national growth, and to structure a general framework. Implementing AM can also result in reduced manufacturing costs, lower shipping costs, and overall lower operating costs since the distributed fabrication of spare parts uses smaller, more automated equipment and can facilitate mass customization. [23] reveal that there is interest in adopting additive manufacturing in spare parts supply chains in order to shorten supply chains, improve response times, and optimize inventory. In the automotive industry, research on the additive manufacturing production of polymer [24] and metal [25] or composite components [17] has continued to widen. Despite the success of AM of polymer composites in the automotive industry, the technology is restricted by the size and number of parts produced because certain components are massive in size and typically need mass production [26]. According to [27], alternative AM technologies that may be utilized for mass production of end-use parts in the automotive industry should be evaluated from many aspects in future research. The selection of a suitable AM process or machine for the fabrication of an end-use product is a crucial aspect of AM design [28]. As can be seen, limited research has been done on additive manufacturing in the automotive industry.

The present study develops a hybrid framework that helps solve the following research questions

- What are the criteria to be considered for the automotive industry within the scope of the additive manufacturing process selection?
- How can the importance weights of the determined evaluation criteria be determined with current approaches?
- What is the most suitable additive manufacturing process for the automotive industry in general?

### 1.1 Motivation and contribution

In this study, the current MCDM methods CRITIC and EDAS were implemented in a Pythagorean fuzzy environment, allowing decision experts to more effectively express and assess their judgments. This paper is significant in that combination of the aforementioned methodologies in terms of evaluation of AM processes and within the perspective of the automotive industry is studied as a contribution to the extant literature.

The primary motivating force behind this research can be seen from two points of view. The first motivation is to establish a novel methodology that is not yet accessible in the literature, and the second one is to put the suggested methodology into practice by addressing a current problem. In this context, the Pythagorean fuzzy CRITIC-EDAS is developed and used to assess different additive manufacturing processes for the automotive industry. The following are the primary contributions of this work, which may also be seen as its innovative points or advantages:

- Our research first gives a broad framework for the selection of additive manufacturing processes in the automotive industry.
- The CRITIC integrated EDAS methodology is provided in a Pythagorean fuzzy setting. The paper describes the Pythagorean fuzzy CRITIC approach, a contemporary and objective weighting method. Combining this weighting approach with the Pythagorean fuzzy EDAS method yields a hybrid model. For an additive manufacturing process in the automotive industry, the combination of these methods is novel.
- Within a comprehensive fuzzy MCDM framework, additive manufacturing processes for the automobile industry are evaluated for the first time.
- The suggested methodology offers a rapid and precise ranking of alternatives while also addressing the need for objective criteria weighting. The suggested approach does not need a distinct weight assignment for each criteria, which may make the outcomes more dependent on the subjective judgements of decision experts.
- Through this study, we intend to enlighten practitioners on the current state of the art in this subject and highlight the significance of MCDM application in determining the optimal additive manufacturing solutions for the automobile industry. In addition, the set of criteria that may be applied to the evaluation of additive manufacturing options within the scope of the study, along with their respective weights, can be viewed as a useful guide for academics and professionals working in this field.
- We undertake a sensitivity analysis for criteria and decision-maker weights to assess the consistency of our methodology. A comparative study is also presented to validate the methodology.

The rest of the paper is organized as follows: Sect. 2 summarizes the related work, Sect. 3 provides the methodology, Sect. 4 illustrates the proposed methodology through a numerical application and Sect. 5 finalizes the paper with conclusion.

## 2 Literature review

Multi-criteria decision making (MCDM) methods are often used to analyze complicated decision-making problems including many and conflicting criteria and a set of alternatives, and are applied to a wide range of problems e.g. prioritization of renewable energy projects [29], assessment of information system governance [30], selection of ideal structural system [31], evaluation of seismic strengths of educational and hospital buildings [32, 33], performance ranking of brands [34], logistics quality analysis [35] and even pandemic readiness analysis [36].

MCDM methods are intended to assist decision-makers in overcoming complex AM process selection issues [37]. In the literature, there are various studies within the scope of AM process assessment. [38] discussed stereolithography (SLA), selective laser sintering (SLS), fused deposition modeling (FDM), and 3D printing (3DP) processes in their study. Researchers using Graph theory and matrix approach (GT MA) and Technique for order of preference by similarity to ideal solution (TOPSIS) methods examined car tail lamp housing, car front grill, and car fuel cap assembly gauge products. Within the scope of the specified products, the processes were evaluated using the criteria of accuracy, surface finish, tensile strength, elongation, heat deflection temperature, part cost, and build time. They claimed that the SLA and SLS processes produced better manufacturing results. Using the fuzzy multicriteria optimization and compromise solution (VIKOR) approach, [39] attempted to choose the AM process most suited to pump impellers. Twenty criteria were used to assess the SLA, SLS, and FDM processes. According to the findings of the research, FDM is the most efficient AM process.

[40] evaluated seven main options using the Analytic Hierarchy Process (AHP) method in their AM process selection study. Technology, materials, size, multicolor feature, resolution, layer thickness, accuracy, speed, power specs, weight, and price were used as evaluation criteria in the study, which examines the 3D printers on the market as well as the process. The machine parts considered in the study are the turbine blade, building scale model and bearing holder. As a result of their analysis, they stated that direct metal laser sintering (DMLS) for turbine blades, colorjet printing (CJP) for building scale models, and FDM processes for bearing holders are suitable. [41] evaluated five different AM processes using Fuzzy AHP and TOPSIS methods. In the study, the laser-induced forward transfer method emerged as the most suitable option. The most important evaluation criteria were material compatibility, geometric complexity, and minimum feature size.

[42] evaluated four different AM processes using the AHP, Fuzzy AHP, and Preference ranking organization method for enrichment evaluation (PROMETHEE) methods in their study. Dimensional accuracy, surface roughness, mechanical properties, process cost, process time, and post-processing criteria were used. The researchers, who made an assessment on a part whose virtual CAD description was presented, chose the photopolymer jetting process as the most suitable solution. [43] evaluated various AM processes under the main criteria of function, cost, and environment. As an industrial case study, they evaluated the drilling grid with AHP and SAW methods in their studies, in which they handled items such as materials, data, and 3D-printer manufacturers in an integrated manner. Using Fuzzy Analytic Network Process (ANP) and Fuzzy TOPSIS methodologies, [44] assessed SLA, SLS, laser engineered net shaping (LENS), and 3DP choices. In order to construct a conceptual framework as opposed to a product-based study, researchers used environmental criteria in addition to the traditional ones. In accordance with the main criteria of mechanical qualities, process capability, manufacturing efficiency, cost, footprint, process emission, and resource use, it was determined that SLA was the most suitable process.

[45] calculated the importance of the evaluation criteria with the help of an expert decision-making group consisting of technical experts and users. They stated that the important criteria to be considered while evaluating AM processes are accuracy, elongation, and maximum build size. Researchers using the AHP method stated that AM processes can be evaluated with different methods in future studies. [46] utilized AHP and cost calculation methodologies. In consideration of many components, such as the exhaust gas duct, the AM process and machine were selected. As a consequence of the assessment, selective laser melting (SLM) was determined as the best appropriate process. [47] compared four different AM processes for a spur gear using the Best-Worst Method (BWM) and proximity indexed value (PIV) methods. The researchers found that the material jetting process was the best choice based on the accuracy of the dimensions, the roughness of the surface, the tensile strength, the percentage of elongation, the heat deflection temperature, the cost of the process, and the time it takes to build.

**Fig 1:**
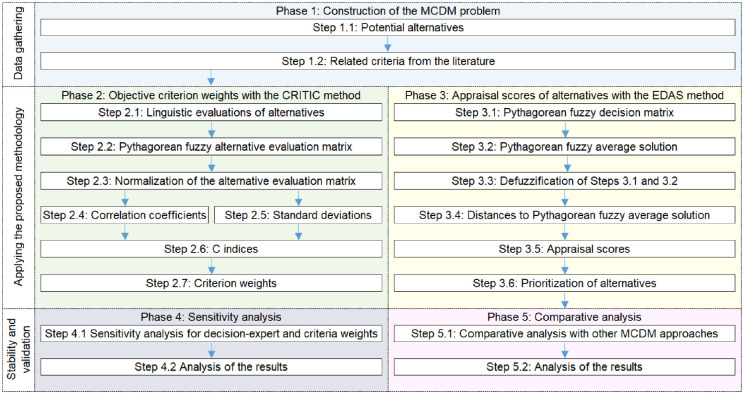
Evaluation of AM processes with MCDM methods in the literature.

[15], using the AHP method, made process selection within the scope of overhangs, bridging, bores and channels, thickness, size, and surface roughness criteria. Vat polymerization, material jetting, extrusion, binder jetting, laser powder bed fusion (LBFM), electron beam melting (EBM), DED, and SL processes were evaluated for gas turbine blade manufacturing. Considering the research criteria and the product examined, it was stated that laser powder bed fusion is the best process to be used. One of the studies conducted in recent years belongs to [37]. Using the certainty pairwise comparison (CPC) and BWM, various AM processes were evaluated for the production of investment casting patterns and an adjustable pasta drying rack. ME, VAT, BJ, MJ, DED, and PBF processes were examined in their studies carried out within the framework of the main criteria of dimensional accuracy, printing speed, surface finish, and cost. As an outcome of the analysis, it was determined that the VAT method yielded successful results. Existing studies are summarized in Fig 1.

Pythagorean fuzzy (PF) sets, represented by membership degree and non-membership degree, are a more effective way of expressing uncertainty [47]. This approach provides more robust, flexible, and ultimately more practical expressions of membership functions by decision-makers [48]. Since it is a relatively new method, studies on Pythagorean fuzzy CRITIC (PF-CRITIC) are also very limited. There are several published papers in the literature regarding the PF-CRITIC, such as assessment of 5G mobile communication technology [47], agriculture crop selection [49], developing business model innovation for sustainability [50], cybersecurity framework prioritization for healthcare organizations [51], supplier selection [52], evaluate the barriers of blockchain technology adoption in sustainable supply chain management [53], cache replacement policy selection in fog computing [54].

EDAS is an MCDM method for identifying solutions from a collection of criteria and alternatives based on the idea that the best alternative is above and farthest from the average solution. Compared to many other decision-making techniques, EDAS is simpler, follows a clear logic that is easy for practitioners to understand, and the mathematical process is relatively basic. Integration of EDAS and Pythagorean fuzzy sets (PF-EDAS) is a recently developed approach that has swiftly attracted attention, having been applied in different fields, including car selection [55], sustainable circular supplier selection in the manufacturing sector [56], evaluating investment projects for highways [57] selecting an optimal drug to treat the Coronavirus ailment [58], supplier selection for coal thermal power plant [59], ship navigation environment safety assessment [60], selection of waste management techniques [61], loan evaluation for banking customers [62], analysis of metaverse integration of freight fluidity assessment solutions [63], evaluation of alternatives for mobility sharing [64] and ranking of use cases for energy blockchain [65].

The aforementioned studies presented their works in a crisp environment, or some authors employed triangular fuzzy sets, which offer a limited area for modeling the fuzziness in the problem. In this study, however, a more extensive fuzzy MCDM is established. Unlike other approaches, the suggested methodology enables calculating criteria weights, ranking alternatives, and modeling the uncertainty in a more comprehensive manner. On the other hand, previous works may offer a limited number of alternatives and criteria. This study provides a satisfactory set of alternatives and criteria with respect to previous approaches.

## 3 Methodology

In this section, the methodology is presented. In Section 3.1, the basic operators of Pythagorean fuzzy sets are summarized, and in Section 3.2, the flowchart and details of the proposed methodology are provided.

### 3.1 Preliminaries of Pythagorean fuzzy sets

Definition, basic operators, aggregation operator and defuzzification operator developed for Pythagorean fuzzy sets [66, 67] are given below:

*Definition* [66, 67].

$$\begin{array}{c} \tilde{X_{P}}=\left\{u,\mu_{\tilde{X_{P}}}\left(u\right),\nu_{\tilde{X_{P}}}\left(u\right)|u\in \cup \right\} \end{array}$$ (1)

where $\mu_{\tilde{X_{P}}}(\text{u}):\cup \to [0,1]$, $\nu_{\tilde{X_{P}}}(\text{u}):\cup \to [0,1]$, and

$$\begin{array}{c} 0\leq \mu_{\tilde{X_{P}}}^{2}\left(u\right)+\nu_{\tilde{X_{P}}}^{2}\left(u\right)\leq 1|\forall u\in \cup \end{array}$$ (2)

where $\mu_{\tilde{X_{P}}}(\text{u})$ and $\nu_{\tilde{X_{P}}}(\text{u})$ are the degrees of membership and non-membership degrees.

$\pi_{\tilde{X_{P}}}(\text{u})$ is the hesitancy of u to $\tilde{X_{P}}$.

$$\begin{array}{c} \pi_{\tilde{X_{P}}}\left(u\right)={\left(1-\mu_{\tilde{X_{P}}}^{2}\left(u\right)-\nu_{\tilde{X_{P}}}^{2}\left(u\right)\right)}^{1/2} \end{array}$$ (3)

*Addition* [66].

$$\begin{array}{c} \begin{array}{c} \tilde{X_{P}}⊕\tilde{Y_{P}}=\left\{{\left(\mu_{\tilde{X_{P}}}^{2}+\mu_{\tilde{Y_{P}}}^{2}-\mu_{\tilde{X_{P}}}^{2}\mu_{\tilde{Y_{P}}}^{2}\right)}^{1/2},\nu_{\tilde{X_{P}}}^{2}\nu_{\tilde{Y_{P}}}^{2}\right\} \end{array} \end{array}$$ (4)

*Multiplication* [66].

$$\begin{array}{c} \begin{array}{c} \tilde{X_{P}}⊗\tilde{Y_{P}}=\mu_{\tilde{X_{P}}}\mu_{\tilde{Y_{P}}},{\left(\nu_{\tilde{X_{P}}}^{2}+\nu_{\tilde{Y_{P}}}^{2}-\nu_{\tilde{X_{P}}}^{2}\nu_{\tilde{Y_{P}}}^{2}\right)}^{1/2}\} \end{array} \end{array}$$ (5)

*Multiplication by a scalar. (*λ *> 0)* [66]

$$\begin{array}{c} \begin{array}{c} \lambda \cdot \tilde{X_{P}}=\left\{{\left(1-{\left(1-\mu_{\tilde{X_{P}}}^{2}\right)}^{\lambda }\right)}^{1/2},\nu_{\tilde{X_{P}}}^{\lambda }\right\} \end{array} \end{array}$$ (6)

*Power of $\tilde{X_{P}}$. (*λ *> 0)* [67]

$$\begin{array}{c} \begin{array}{c} {\tilde{X_{P}}}^{\lambda }=\left\{\mu_{\tilde{X_{P}}}^{\lambda },{\left(1-{\left(1-\nu_{\tilde{X_{P}}}^{2}\right)}^{\lambda }\right)}^{1/2}\right\} \end{array} \end{array}$$ (7)

*Pythagorean weighted geometric mean operator PWGM* [68].

$$\begin{array}{c} \begin{array}{c} {{PWGM}_{w}\left(\tilde{X_{P1}},\ldots ,\tilde{X_{Pn}}\right)={\tilde{X_{P1}}}^{w1}+{\tilde{X_{P2}}}^{w2}+\ldots .+\tilde{X_{Pn}}}^{w_{n}}=\\ \left\{\prod_{i=1}^{n}\mu_{\tilde{X_{Pi}}}^{w_{i}},{\left[1-\prod_{i=1}^{n}{\left(1-\nu_{\tilde{X_{i}}}^{2}\right)}^{w_{j}}\right]}^{1/2}\right\} \end{array} \end{array}$$ (8)

where $w=\left(w_{1},w_{2},\ldots ,w_{n}\right);w_{i}\in \left[0,1\right];\sum_{i=1}^{n}w_{i}=1$

*Defuzzification operator i.e. score function* [69].

$$\begin{array}{c} S\left(\tilde{X_{P}}\right)=\mu_{\tilde{X_{P}}}^{2}-\nu_{\tilde{X_{P}}}^{2} \end{array}$$ (9)

*Normalized Euclidean distance D* [70].

$$\begin{array}{c} D\left(\tilde{X_{P}},\tilde{Y_{P}}\right)=\sqrt{\frac{1}{2n}\sum_{i=1}^{n}{\left(\mu_{\tilde{X_{P}}}-\mu_{\tilde{Y_{P}}}\right)}^{2}+{\left(\nu_{\tilde{X_{P}}}-\nu_{\tilde{Y_{P}}}\right)}^{2}+{\left(\pi_{\tilde{X_{P}}}-\pi_{\tilde{Y_{P}}}\right)}^{2}} \end{array}$$ (10)

### 3.2 Proposed methodology

**Fig 2:**
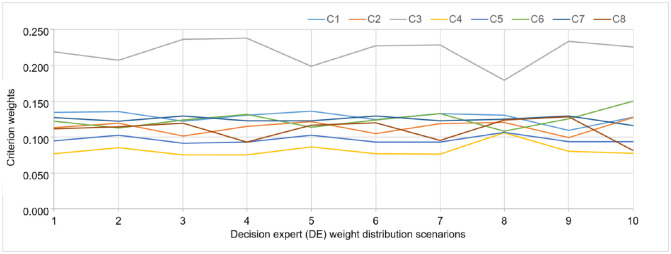
Flowchart of the proposed methodology.

Prior to describing the proposed methodology in detail, the flowchart is given in Fig 2 to provide the reader a general notion of the methodology.

**Phase 1.** Construction of the MCDM problem.

**Phase 2.** Evaluate criterion weights with Pythagorean fuzzy CRITIC.

*Step 2.1* The decision-experts evaluate the alternatives with respect to the criteria by utilizing the linguistic terms that is given in Table 1.

**Table 1:** Linguistic scale for Pythagorean fuzzy numbers [71].

Linguistic term	Pythagorean fuzzy number (*μ*, *ν*)
Very Low (VL)	(0.25, 0.90)
Low (L)	(0.30, 0.85)
Medium Low (ML)	(0.35, 0.75)
Medium (M)	(0.45, 0.65)
High (H)	(0.70, 0.35)
Very High (VH)	(0.80, 0.30)

*Step 2.2* Linguistic evaluations of decision-experts are converted to Pythagorean fuzzy numbers through Table 1. Then, all matrices are aggregated to obtain one unique collective matrix which we call Pythagorean fuzzy alternative assessment matrix $\tilde{M}$. The structure of $\tilde{M}$ is given in Eq 11.

$$\begin{array}{c} {\tilde{M}}_{mxn}=[\begin{array}{cccc} {\tilde{r}}_{11} & {\tilde{r}}_{12} & \ldots & {\tilde{r}}_{1n}\\ {\tilde{r}}_{21} & {\tilde{r}}_{22} & \ldots & {\tilde{r}}_{2n}\\ \ldots & \ldots & \ldots \\ {\tilde{r}}_{m1} & {\tilde{r}}_{m2} & \ldots & {\tilde{r}}_{mn} \end{array}]\; \end{array}$$ (11)

where ${\tilde{r}}_{ij}=\mu_{ij},\nu_{ij}$ are the elements of $\tilde{M}$; the evaluation of alternative *X*_*i*_(*i* = 1, 2, …, *m*) with respect to criterion *C*_*j*_(*j* = 1, 2, …, *n*) is donated by $\tilde{M}=C_{j}{\left(X_{i}\right)}_{mxn}$ and *μ*_*ij*_ and *ν*_*ij*_ are the membership and non-membership degrees for the *i*th alternative and *j*th criterion.

*Step 2.3* $\tilde{M}$ is normalized by utilizing Eqs 12 and 13 for positive (benefit) and negative (cost) attributes respectively.

$$\begin{array}{c} x_{ij}=\frac{{\tilde{r}}_{ij}-{\tilde{r}}_{i}^{-}}{{\tilde{r}}_{i}^{+}-{\tilde{r}}_{i}^{-}},i=1,2,\ldots ,m;j=1,2,\ldots ,n \end{array}$$ (12)

$$\begin{array}{c} x_{ij}=\frac{{\tilde{r}}_{ij}-{\tilde{r}}_{i}^{+}}{{\tilde{r}}_{i}^{-}-{\tilde{r}}_{i}^{+}},i=1,2,\ldots ,m;j=1,2,\ldots ,n \end{array}$$ (13)

where *x*_*ij*_ is the normalized value of *r*_*ij*_; $r_{i}^{+}=max\left(r_{1},r_{2},\ldots ,r_{m}\right)$; $r_{i}^{-}=min\left(r_{1},r_{2},\ldots ,r_{m}\right)$.

$r_{i}^{+}$ and $r_{i}^{-}$ are obtained based on the defuzzified values by utilizing Eq 10.

Normalized Euclidean distance that is given in Eq 10 is used to obtained the nominator and denominator parts of the above equations.

*Step 2.4* The correlation coefficient *ρ*_*jk*_ between each attribute pair is calculated by utilizing Eq 14.

$$\begin{array}{c} \begin{array}{c} \rho_{jk}=\frac{\sum_{i=1}^{m}\left(x_{ij}-{\bar{x}}_{j}\right)\left(x_{ik}-{\bar{x}}_{k}\right)}{\sqrt{\sum_{i=1}^{m}{\left(x_{ij}-{\bar{x}}_{j}\right)}^{2}\sum_{i=1}^{m}{\left(x_{ik}-{\bar{x}}_{k}\right)}^{2}}} \end{array} \end{array}$$ (14)

where ${\bar{x}}_{j}$ and ${\bar{x}}_{k}$ are the mean values of *j*th and *k*th attributes, and ${\bar{x}}_{j}$ is obtained by utilizing Eq 15. ${\bar{x}}_{k}$ is also obtained in the same way.

$$\begin{array}{c} {\bar{x}}_{j}=\frac{1}{n}\sum_{j=1}^{n}x_{ij},i=1,2,\ldots ,m \end{array}$$ (15)

*Step 2.5* The standard deviation *σ*_*j*_ of each criterion is calculated as given in Eq 16.

$$\begin{array}{c} \sigma_{j}=\sqrt{\frac{1}{n-1}\sum_{j=1}^{n}{\left(x_{ij}-{\bar{x}}_{j}\right)}^{2})},i=1,2,\ldots ,m \end{array}$$ (16)

*Step 2.6* The *C*_*j*_ index of each criterion is calculated as given in Eq 17.

$$\begin{array}{c} C_{j}=\sigma_{j}\sum_{k=1}^{n}\left(1-\rho_{jk}\right),j=1,2,\ldots ,n \end{array}$$ (17)

*Step 2.7* Criterion weights *w*_*j*_ are obtained as given in Eq 18.

$$\begin{array}{c} w_{j}=\frac{C_{j}}{\sum_{j=1}^{n}C_{j}} \end{array}$$ (18)

**Phase 3** Rank the candidates with Pythagorean fuzzy EDAS.

*Step 3.1* Pythagorean fuzzy decision matrix $\tilde{D}$ is obtained by multiplying $\tilde{M}$ by the criterion weights *w*_*j*_ calculated with the CRITIC approach in the previous phase. Pythagorean fuzzy multiplication by a scalar function that is given in Eq 6 is used for this task.

*Step 3.2* The average solution ${\tilde{X}}^{*}$ is obtained by utilizing Pythageoran fuzzy geometric mean operator that is given in Eq 8.

*Step 3.3* Determine the positively positioned alternatives and negatively positioned alternatives based on the crisp values calculated by utilizing the defuzzification operator that is given in Eq 10 For each criterion, the alternatives with a higher score value than the average solution is assigned as positively positioned alternative. In the same way, the alternatives with a lower score value than the average solution is assigned as negatively positioned alternative.

*Step 3.4* Calculate the positive and negative distances of each alternative to average solution ${\tilde{X}}^{*}$ by utilizing Eq 10. Note that the theory of the EDAS technique suggests that the best alternative is the one that has maximum total positive distance to average solution *PDA* and minimum total negative distance to average solution *NDA*.

*Step 3.5* Closeness ratio *CR* i.e. appraisal score for each candidate is obtained as in Eq 19. Rank all candidates according to the descending values of their appraisal scores. The candidate with the highest appraisal score is the most critical.

$$\begin{array}{c} CR=\frac{PDA({\tilde{X}}_{i},{\tilde{X}}^{*})}{PDA\left({\tilde{X}}_{i},{\tilde{X}}^{*}\right)+NDA\left({\tilde{X}}_{i},{\tilde{X}}^{*}\right)} \end{array}$$ (19)

*Step 3.6* The candidates are ranked according to appraisal scores. The one with highest appraisal score is the most critical.

**Phase 4** Sensitivity analysis.

**Phase 5** Comparative analysis.

## 4 Application

In this section, the application of the proposed methodology is exemplified by an issue involving the selection of an additive manufacturing process for the automotive industry. To do this, seven additive manufacturing processes are assessed based on eight literature-derived criteria. The decision-experts are industry specialists with comparable levels of expertise; therefore, their opinions carry equal weight (1/3) This section is structured as follows: In Section 4.1, the criteria and alternatives are described in depth. The numerical solution to the problem is presented in Section 4.2. Section 4.3 offers sensitivity analysis for criteria and decision-expert weights, whereas Section 4.4 performs a comparison analysis.

### 4.1 Alternatives and criteria

The descriptions of alternatives and criteria are given below.

*A1 Vat photopolymerization.* This is one of the most widely used procedures for 3D printing. In this method, the use of ultraviolet light helps to solidify liquid light-curable resin by forming chains between the molecules of the resin, which then cause the molecules to cross-link with one another [72].

*A2 Material extrusion.* In the additive manufacturing process known as material extrusion, material is distributed selectively via a nozzle in order to create a desired shape. Fused deposition modeling, is one of the most prevalent processes for extruding materials. This method requires high working temperatures to successfully fuse materials [73].

*A3 Material jetting.* The method of material jetting is one kind of additive manufacturing that includes applying UV light and droplets of material over a specific region in order to selectively cure the components. The material may be jetted either on demand or constantly, depending on the nature of the item that is to be manufactured using the 3D printer [74].

*A4 Binder jetting.* This is a two-phase additive manufacturing process that uses powder beds. To create the cross section of each layer during printing, liquid binding agents are first selectively put into the powder bed using the CAD model as a guide. The printed portion is next subjected to curing and depowdering. The printed pieces are then put in a uniform heat environment throughout the debinding and sintering operations to improve their mechanical integrity [75, 76].

*A5 Powder bed fusion.* This method is used to fabricate complicated lattice products from metallic alloys. Difficulties exist in the general use of this approach owing to the propensity for powder bed fusion produced components to acquire flaws and poorer physical qualities, which may lead to failure in these particular applications. These flaws include a poor surface finish, an increase in porosity, delamination, and cracking, which result in lower mechanical performance and poor geometric conformance [77, 78].

*A6 Direct energy deposition.* With the use of a closed-loop control system to preserve dimensional precision and material integrity, direct energy deposition is a method that creates completely dense, functioning metal components by depositing metal particles. By reconstructing individual worn-out or broken components, this approach allows for both component manufacturing and repair. Its high reflectivity presents one of the main difficulties since it results in a poor rate of energy absorption, which encourages consolidation flaws such as pores, fractures, and unmelted regions [79].

*A7 Sheet lamination.* The technique of sheet lamination is based on the fabrication of a product by stacking and laminating sheets of different materials. For plastics or other chemically bonded materials, such as brazing or ultrasonic welding, each layer is laminated. After fabrication is finished, the extra area is taken away layer by layer. The fabrication of laminated objects and ultrasonic additive manufacturing are completely dependent on sheet lamination [80].

*C1 Surface roughness.* This is commonly regarded as one of the most essential quality criteria, along with surface attractiveness, resistance to corrosion, increased fatigue behavior, surface concerns, and adequate purity of a significant surface. The mechanism of product surface roughness generation is very dynamic, complicated, and manufacturing process-dependent [81].

*C2 Dimensional accuracy.* In any manufacturing process, dimensional accuracy is essential since it indicates how closely a dimension of the manufactured piece matches the nominal dimension of the planned component [82].

*C3 Build volume.* It provides the largest print size possible. Build volume is considered to be one of the most critical challenges presented by additive manufacturing technology. Huge servings are often reduced in size or broken into smaller sections, both of which require a significant investment of time and effort. In most cases, reducing the size of the model to a more manageable proportion is neither feasible nor productive. When adhesives are utilized, the assembly of component parts suffers a loss of strength. On the other hand, when mechanical fasteners are used, the assembly grows in size and thickness [83].

*C4 Mechanical resistance.* The use of products or components in various applications is significantly influenced by the strength and stiffness of the materials used. Typically, the physical and mechanical qualities of the materials are taken into account when setting process parameters for additive manufacturing. For instance, in aerospace applications, the strength-to-weight ratio of the component is crucial in the development of better functioning products [84].

*C5 Cost of the process.* This criterion includes the total initial cost and support material costs. The cost of additive manufacturing can be divided into two categories. The first is to compare additive manufacturing technologies to conventional procedures like injection molding and machining. These types of analyses are carried out to determine the conditions under which additive manufacturing is cost-effective. The second category is identifying resource use at different additive manufacturing process phases. The objective of this sort of study is to determine when and where resources are spent and if resource consumption may be reduced [85].

*C6 Print speed.* Speed of printing is a crucial performance aspect in 3D printing and a major obstacle that, in certain situations, precludes it from becoming a feasible method of production. Even though 3D printing is often quicker than traditional production, it may still take hours or even days to make a product [86].

*C7 Thermal stability.* The resistance of a material to irreversible changes in its properties brought about purely by heat is referred to as its “thermal stability.” The capacity of a product to maintain its temperature stability may be an important factor to consider when deciding whether or not it is appropriate for a certain application [87].

*C8 Chemical resistance.* Along with having high mechanical qualities, 3D printed products also need to have good chemical resistance. Making coolant inlet tubes, for instance, in the automotive industry, which transfer coolant fluids to the cooling system of the car and require high mechanical quality and structural stability in continuous exposure to the chemicals in the car coolant at high temperatures, is one potential application [88].

### 4.2 Numerical solution

*C*riterion weights with Pythagorean fuzzy CRITIC. *Step 2.1* The linguistic evaluations of seven AM processes (*A*1, …, *A*7) with regard to seven criteria (*C*1, …, *C*7) as decided by three decision experts (*DE*1, …, *DE*3) are shown in Table 2.

**Table 2:** Linguistic evaluations of alternatives.

	Alternative	C1	C2	C3	C4	C5	C6	C7	C8
DM1	A1	VH	H	ML	L	ML	VH	L	M
	A2	L	M	MH	ML	VL	ML	MH	M
	A3	H	H	M	MH	H	ML	H	H
	A4	MH	H	MH	MH	MH	M	MH	MH
	A5	MH	H	MH	MH	MH	H	H	H
	A6	MH	ML	H	H	VH	M	VH	VH
	A7	L	L	MH	EL	VL	ML	EL	EL
DM2	A1	H	MH	ML	ML	MH	MH	VL	L
	A2	M	MH	VH	VL	ML	VL	MH	ML
	A3	MH	ML	VL	H	VH	ML	H	H
	A4	H	VH	MH	MH	MH	M	M	MH
	A5	M	M	VH	M	ML	MH	H	H
	A6	VH	M	H	H	M	MH	VH	MH
	A7	VL	EL	MH	EL	EL	ML	VL	M
DM3	A1	MH	VH	ML	M	M	VH	L	ML
	A2	EL	EL	MH	ML	VL	EL	MH	M
	A3	H	H	ML	ML	H	ML	H	H
	A4	EL	H	M	VL	H	M	MH	L
	A5	EL	ML	ML	MH	M	ML	L	H
	A6	MH	MH	VH	H	M	M	MH	VH
	A7	EL	L	M	M	ML	EL	EL	L

*Step 2.2* Linguistic expressions of three decision-experts are converted to Pythagorean fuzzy numbers and aggregated as in Table 3.

**Table 3:** Pythagorean fuzzy alternative assessment matrix.

	C1	C2	C3	C4	C5	C6	C7	C8
A1	(0.70;0.40)	(0.70;0.40)	(0.35;0.75)	(0.36;0.77)	(0.46;0.65)	(0.73;0.38)	(0.28;0.87)	(0.36;0.77)
A2	(0.27;0.87)	(0.34;0.81)	(0.66;0.45)	(0.31;0.82)	(0.28;0.87)	(0.24;0.89)	(0.60;0.50)	(0.41;0.69)
A3	(0.66;0.41)	(0.56;0.55)	(0.34;0.80)	(0.53;0.58)	(0.73;0.33)	(0.35;0.75)	(0.70;0.35)	(0.70;0.35)
A4	(0.40;0.77)	(0.73;0.33)	(0.55;0.56)	(0.45;0.72)	(0.63;0.46)	(0.45;0.65)	(0.55;0.56)	(0.48;0.68)
A5	(0.34;0.81)	(0.48;0.63)	(0.55;0.58)	(0.55;0.56)	(0.46;0.65)	(0.53;0.58)	(0.53;0.63)	(0.70;0.35)
A6	(0.66;0.45)	(0.46;0.65)	(0.73;0.33)	(0.70;0.35)	(0.55;0.57)	(0.50;0.61)	(0.73;0.38)	(0.73;0.38)
A7	(0.22;0.91)	(0.24;0.90)	(0.55;0.56)	(0.22;0.91)	(0.24;0.89)	(0.26;0.86)	(0.18;0.94)	(0.27;0.87)

*Steps 2.3 and 2.4* Correlation coefficients are calculated as in Table 4.

**Table 4:** Correlation coefficients.

	C1	C2	C3	C4	C5	C6	C7	C8
C1	1.000	0.620	-0.463	0.608	0.698	0.630	0.304	0.445
C2	0.620	1.000	-0.452	0.423	0.829	0.778	0.256	0.313
C3	-0.463	-0.452	1.000	0.104	-0.420	-0.284	0.193	-0.001
C4	0.608	0.423	0.104	1.000	0.699	0.485	0.705	0.921
C5	0.698	0.829	-0.420	0.699	1.000	0.548	0.569	0.652
C6	0.630	0.778	-0.284	0.485	0.548	1.000	-0.085	0.260
C7	0.304	0.256	0.193	0.705	0.569	-0.085	1.000	0.802
C8	0.445	0.313	-0.001	0.921	0.652	0.260	0.802	1.000

*Step 2.5, 2.6 and 2.7* Standard deviation *σ*_*j*_, *C* index and criterion weights *w*_*j*_ are calculated as in Table 5.

**Table 5:** Standard deviation *σ*_*j*_, *C* index and criterion weights *w*_*j*_.

	C1	C2	C3	C4	C5	C6	C7	C8
j	0.438	0.363	0.356	0.341	0.375	0.354	0.405	0.419
*C* index	1.823	1.535	2.965	1.042	1.284	1.652	1.723	1.510
*w*_*j*_	0.135	0.113	0.219	0.077	0.095	0.122	0.127	0.112

*Phase 3* Ranking of alternative processes with Pythagorean fuzzy EDAS.

In Table 6, fuzzy decision matrix and average solution are expressed. In Table 7, the appraisal score values of the alternatives and the final ranking were presented.

**Table 6:** Pythagorean fuzzy decision matrix and average solution.

	C1	C2	C3	C4	C5	C6	C7	C8
A1	(0.29;0.88)	(0.27;0.90)	(0.17;0.94)	(0.10;0.98)	(0.15;0.96)	(0.30;0.89)	(0.10;0.98)	(0.12;0.97)
A2	(0.10;0.98)	(0.12;0.98)	(0.34;0.84)	(0.09;0.98)	(0.09;0.99)	(0.08;0.99)	(0.24;0.92)	(0.14;0.96)
A3	(0.27;0,89)	(0.20;0.93)	(0.16;0.95)	(0.16;0.96)	(0.26;0.90)	(0.13;0.97)	(0.29;0.87)	(0.27;0.89)
A4	(0.15;0.97)	(0.29;0.88)	(0.27;0.88)	(0.13;0.98)	(0.22;0.93)	(0.17;0.95)	(0.21;0.93)	(0.17;0.96)
A5	(0.13;0.97)	(0.17;0.95)	(0.28;0.89)	(0.16;0.96)	(0.15;0.96)	(0.20;0.94)	(0.20;0.94)	(0.27;0.89)
A6	(0.27;0.90)	(0.16;0.95)	(0.39;0.79)	(0.22;0.92)	(0.18;0.95)	(0.18;0.94)	(0.30;0.89)	(0.28;0.90)
A7	(0.08;0.99)	(0.08;0.99)	(0.27;0.88)	(0.06;0.99)	(0.07;0.99)	(0.09;0.98)	(0.06;0.99)	(0.09;0.98)
A *	(0.17;0.96)	(0.17;0.95)	(0.26;0.89)	(0.12;0.97)	(0.15;0.96)	(0.15;0.96	(0.18;0.95)	(0.18;0.95)

**Table 7:** PDA, NDA, appraisal score and final ranking of alternatives.

	PDA	NDA	Appraisal score	Final ranking
A1	0.078	0.049	0.615	5
A2	0.038	0.055	0.411	6
A3	0.091	0.039	0.698	4
A4	0.057	0.012	0.820	2
A5	0.047	0.014	0.764	3
A6	0.097	0.003	0.972	1
A7	0.007	0.091	0.071	7

As a result of the analysis, in line with the opinions of the experts, “A6- Direct energy deposition” has been identified as the most appropriate alternative.

### 4.3 Sensitivity analysis

Sensitivity analysis is often used to evaluate the effectiveness of an MCDM procedure by measuring the output in response to varying inputs [89]. In this study, the effect of the changes in both criterion and decision expert weights on the output of the proposed methodology are examined. The sensitivity analysis for criterion weights is carried out by generating different scenarios based on the original scenario. For this purpose, the initial scenario *w*1 is systematically shifted, and six more scenarios *w*2, *w*3, *w*4, *w*6, and *w*7 are generated as shown in Table 8.

**Table 8:** Criteria weight distribution scenarios.

	C1	C2	C3	C4	C5	C6	C7	C8
w1	0.135	0.113	0.219	0.077	0.095	0.122	0.127	**0.112**
w2	0.113	0.219	0.077	0.095	0.122	0.127	**0.112**	0.135
w3	0.219	0.077	0.095	0.122	0.127	**0.112**	0.135	0.113
w4	0.077	0.095	0.122	0.127	**0.112**	0.135	0.113	0.219
w5	0.095	0.122	0.127	**0.112**	0.135	0.113	0.219	0.077
w6	0.122	0.127	**0.112**	0.135	0.113	0.219	0.077	0.095
w7	0.127	**0.112**	0.135	0.113	0.219	0.077	0.095	0.122
w8	**0.112**	0.135	0.113	0.219	0.077	0.095	0.122	0.127

In Table 9, the numbers in parentheses next to the appraisal scores represent the ranking value. When the results are analyzed, it can be said that the appraisal score values vary in various criterion weight distribution scenarios, however the ranks remain the same, and the suggested methodology yields balanced outcomes in response to the criterion weight changes.

**Table 9:** Appraisal scores and final rankings of candidates for different criterion weight distribution scenarios.

	w1	w2	w3	w4	w5	w6	w7	w8
A1	0.615 (5)	0.664 (5)	0.651 (5)	0.601 (5)	0.592 (5)	0.691 (5)	0.623 (5)	0.619 (5)
A2	0.411 (6)	0.328 (6)	0.355 (6)	0.368 (6)	0.416 (6)	0.314 (6)	0.347 (6)	0.373 (6)
A3	0.698 (4)	0.790 (4)	0.790 (3)	0.755 (4)	0.760 (4)	0.745 (4)	0.762 (4)	0.754 (4)
A4	0.820 (2)	0.850 (2)	0.791 (2)	0.808 (3)	0.848 (2)	0.829 (2)	0.833 (2)	0.833 (2)
A5	0.764 (3)	0.791 (3)	0.732 (4)	0.844 (2)	0.780 (3)	0.781 (3)	0.774 (3)	0.800 (3)
A6	0.972 (1)	0.956 (1)	0.978 (1)	0.975 (1)	0.971 (1)	0.969 (1)	0.972 (1)	0.972 (1)
A7	0.071 (7)	0.039 (7)	0.044 (7)	0.050 (7)	0.050 (7)	0.050 (7)	0.053 (7)	0.048 (7)

Furthermore, for a more extensive sensitivity analysis, the impact of changing decision-expert weight distributions on the outcomes is examined. In this setting, 10 different decision expert weight distribution scenarios are generated as shown in Table 10.

**Table 10:** Decision-expert (DE) weight distributions (DE1; DE2; DE3).

1	2	3	4	5	6	7	8	9	10
(0.33; 0.33; 0.33)	(0.20; 0.20; 0.40)	(0.20; 0.40; 0.20)	(0.25; 0.25; 0.50)	(0.25; 0.50; 0.25)	(0.50; 0.25; 0.25)	(0.15; 0.15; 0.70)	(0.15; 0.15; 0.70)	(0.15; 0.70; 0.15)	(0.70; 0.15; 0.15)

**Fig 3:**
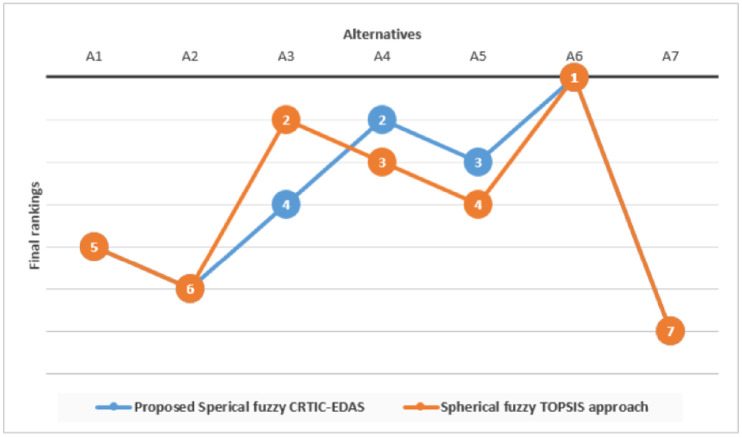
Criterion weights for different decision-expert weight distribution scenarios.

Criterion weights calculated by the CRITIC phase of the methodology in this manner is shown in Fig 3.

In accordance with various decision expert weight distribution scenarios, it can be seen in in Fig 3 that the criterion weights do not generally alter. The third criterion remains the most significant criterion, whilst the fourth criterion remains the least important criterion. It might be claimed that other criteria typically maintain their position.

Appraisal scores and final rankings of candidates for different decision-expert weight distribution scenarios obtained in the EDAS phase of the methodology are given in Table 11.

**Table 11:** Appraisal scores and final rankings of candidates for different decision expert weight distribution scenarios.

	1	2	3	4	5	6	7	8	9	10
A1	0.615 (5)	0.649 (5)	0.553 (5)	0.604 (5)	0.660 (5)	0.565 (5)	0.616 (5)	0.701 (5)	0.505 (5)	0.615 (5)
A2	0.411 (6)	0.377 (6)	0.445 (6)	0.372 (6)	0.387 (6)	0.455 (6)	0.381 (6)	0.369 (6)	0.496 (6)	0.335 (6)
A3	0.698 (4)	0.739 (2)	0.636 (4)	0.697 (4)	0.748 (2)	0.646 (4)	0.706 (4)	0.800 (2)	0.601 (4)	0.722 (4)
A4	0.820 (2)	0.706 (3)	0.890 (2)	0.878 (2)	0.718 (3)	0.903 (2)	0.892 (2)	0.640 (4)	1.000 (2)	1.000 (1)
A5	0.764 (3)	0.689 (4)	0.780 (3)	0.817 (3)	0.706 (4)	0.789 (3)	0.827 (3)	0.643 (3)	0.825 (3)	0.942 (2)
A6	0.972 (1)	1.000 (1)	0.970 (1)	0.923 (1)	1.000 (1)	1.000 (1)	0.927 (1)	1.000 (1)	1.000 (1)	0.856 (3)
A7	0.071 (7)	0.046 (7)	0.086 (7)	0.072 (7)	0.049 (7)	0.090 (7)	0.075 (7)	0.023 (7)	0.110 (7)	0.079 (7)

In a similar vein, it can be noticed that in various decision expert weight distribution scenarios, the alternative rankings are typically maintained. It is apparent that the sixth alternative is ranked first in nine out of ten scenarios, while the seventh alternative is ranked last in every single scenario. In this context, it is possible to conclude that the proposed methodology is typically stable across a variety of criterion and decision expert weight distribution scenarios.

### 4.4 Comparative analysis

In this subsection, a comparative study is performed to test and validate the applicability of the proposed methodology. In this context, the identical problem is addressed via Pythagorean fuzzy TOPSIS method [90], by using the criterion weights that are already calculated with the CRITIC phase of the proposed methodology.

For a comprehensive understanding of the comparative analysis, the steps of the TOPSIS approach are outlined below and following these steps, the solution to the problem is presented.

After obtaining Pythagorean fuzzy decision matrix $\tilde{D}$, TOPSIS approach continues as folows:

$\tilde{D}$ is defuzzified for calculating positive *X*^+^ and negative ideal *X*^−^ solutions. Since crisp form of fuzzy numbers can be used for ranking fuzzy numbers, it is also convenient to obtain positive and negative ideal solutions. ${\tilde{X}}^{+}$ has the highest score values for each criterion and ${\tilde{X}}^{-}$ has the lowest. Defuzzification operator that is given in Eq 10 is used to calculate crisp values.

Pythagorean fuzzy positive ${\tilde{X}}^{+}$ and negative ideal solutions *X*^−^ are determined based on the defuzzified as in Eqs 20 and 21 respectively.

$$\begin{array}{c} {\tilde{X}}^{+}=C_{j},max<S\left(C_{j}\left({\tilde{X}}_{i}\right)>\right|j=1,2,\ldots ,n \end{array}$$ (20)

$$\begin{array}{c} {\tilde{X}}^{-}=C_{j},min<S\left(C_{j}\left({\tilde{X}}_{i}\right)>\right|j=1,2,\ldots ,n \end{array}$$ (21)

The distances of each alternative to ${\tilde{X}}^{+}$ and ${\tilde{X}}^{-}$ are calculated as in Eqs 22 and 23. According to theory of TOPSIS, the best alternative is the one that is closest to the ${\tilde{X}}^{+}$ and farthest from the ${\tilde{X}}^{-}$.

$$\begin{array}{c} \begin{array}{c} d\left({\tilde{X}}_{i},{\tilde{X}}^{+}\right)=\sqrt{\frac{1}{2n}\sum_{j=1}^{n}{\left(a_{ij}-a_{j}^{+}\right)}^{2}+{\left(b_{ij}-b_{j}^{+}\right)}^{2}+{\left(c_{ij}-c_{j}^{+}\right)}^{2}} \end{array} \end{array}$$ (22)

$$\begin{array}{c} \begin{array}{c} d\left({\tilde{X}}_{i},{\tilde{X}}^{-}\right)=\sqrt{\frac{1}{2n}\sum_{j=1}^{n}{\left(a_{ij}-a_{j}^{-}\right)}^{2}+{\left(b_{ij}-b_{j}^{-}\right)}^{2}+{\left(c_{ij}-c_{j}^{-}\right)}^{2}} \end{array} \end{array}$$ (23)

where *a*_*i*_, *b*_*i*_ and *c*_*i*_ are the membership, non-membership and hesitancy degrees for the *i*th candidate and *j*th criterion; *n* is the number of criteria, and $a_{j}^{+}$, $b_{j}^{+}$ and $c_{j}^{+}$ are the parameters of ${\tilde{X}}^{+}$. In the same way $a_{j}^{-}$, $b_{j}^{-}$ and $c_{j}^{-}$ are the parameters ${\tilde{X}}^{-}$.

Closeness ratio *CR* i.e. appraisal score for each candidate is obtained as in Eq 24. Rank all candidates according to the descending values of their appraisal scores. The candidate with the highest appraisal score is the most critical.

$$\begin{array}{c} CR=\frac{d({\tilde{X}}_{i},{\tilde{X}}^{+})}{d\left({\tilde{X}}_{i},{\tilde{X}}^{+}\right)+d\left({\tilde{X}}_{i},{\tilde{X}}^{-}\right)} \end{array}$$ (24)

Pythagorean fuzzy positive *A*^+^ and negative ideal *A*^−^+ solutions intrinsic to the TOPSIS methodology is shown in Table 12.

**Table 12:** Pythagorean fuzzy positive *A*^+^ and negative ideal *A*^−^ solutions.

	C1	C2	C3	C4	C5	C6	C7	C8
A+	(0.29;0.88)	(0.29;0.88)	(0.39;0.79)	(0.22;0.92)	(0.26;0.90)	(0.30;0.89)	(0.29;0.87)	(0.29;0.87)
A -	(0.08;0.99)	(0.08;0.99)	(0.16;0.95)	(0.06;0.99)	(0.07;0.99)	(0.08;0.99)	(0.06;0.99)	(0.10;0.98)

**Fig 4:**
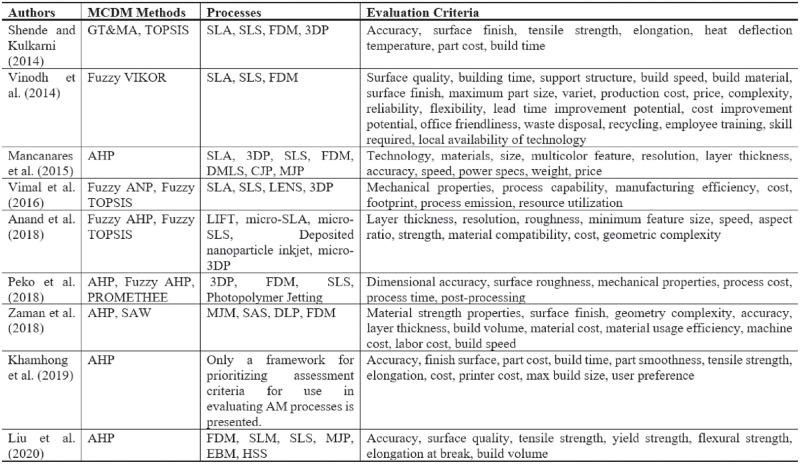
Comparative analysis results.

Following the principles of the TOPSIS methodology, distances of alternatives to *A*^+^ and *A*^−^; corresponding appraisal scores and final rankings are determined as shown in Table 13. Fig 4 displays a graphical representation of the alternative rankings derived by the proposed methodology and the TOPSIS method.

**Table 13:** Comparative analysis results.

	Distance to *A*^+^	Distance to *A*^−^	Appraisal score	Final ranking
A1	0.3143	0.3326	0.3695	5
A2	0.3166	0.3366	0.3689	6
A3	0.3113	0.3380	0.3770	2
A4	0.3110	0.3352	0.3761	3
A5	0.3114	0.3341	0.3750	4
A6	0.3068	0.3374	0.3847	1
A7	0.3213	0.3347	0.3619	7

It can be noticed that results from a comparative research are consistent with the suggested approach.

The outcomes of comparative analysis are also subjected to statistical analysis. In order to determine whether there is a substantial rank correlation between two sets of values, we use the Spearman’s rank correlation technique, which is described in Eq 25.

$$\begin{array}{c} \begin{array}{c} \rho_{xy}=1-\frac{6}{m(m^{2}-1)}\sum_{i=1}^{m}{\left(t_{i}^{x}-t_{i}^{y}\right)}^{2} \end{array} \end{array}$$ (25)

where *x* and *y* denote the *x*th and *y*th MCDM methods, respectively; *m* is the number of alternatives, $t_{i}^{x}$ and $t_{i}^{y}$ are the rankings obtained from *x*th and *y*th MCDM methods. The coefficient *ρ* measures the degree of consistency between them. The correlation coefficient value of the rankings of the proposed methodology and the TOPSIS-based method is 0.89. These findings indicate a strong correlation between the outcomes of the two methodologies.

## 5 Conclusion

Additive manufacturing has considerable promise for the creation of innovative products and processes in a variety of industries, including the automotive industry. However, given the wide range of alternatives to additive manufacturing that are now accessible, each with its own distinctive features, choosing the best one has become essential for pertinent agencies. In this study, the problem of selecting the best additive manufacturing process for the automotive industry is addressed through a novel MCDM methodology by involving multiple experts, criteria and alternatives.

In this context, the traditional CRITIC and EDAS approaches, both of which have previously been recognized in the literature for a range of problems, are integrated in a Pythagorean fuzzy atmosphere. The Pythagorean fuzzy sets provide complete modeling of the problem’s uncertainty; the CRITIC enables the user to objectively establish the criteria; and the EDAS rates the alternatives. The approach described is exemplified with a numerical example for the automotive industry, sensitivity and comparative analyses are provided to show the stability and validity of the results.

Using the suggested Pythagorean fuzzy CRITIC EDAS technique, seven additive manufacturing process alternatives are evaluated based on eight literature-derived criteria. According to the provided numerical example, “build volume” is considered to be the most essential evaluation factor for additive manufacturing alternatives. On the other hand, the EDAS phase of the methodology ranks the alternatives based on their assessment scores, and “direct energy deposition” is selected as the optimal process. This study offers practitioners with a useful instrument for identifying the additive manufacturing solution most suited for the automotive sector.

In the future, the suggested technique may be used in other projects to address a range of additional problems e.g., supply chain model evaluation [91], interval-valued Pythagorean fuzzy sets may be used to increase the fuzziness modeling capacity of the methodology; other fuzzy sets, such as neutrosophic sets, may be used with the CRITIC integrated EDAS technique; other possible variants of this technique, such as WASPAS or DEMATEL integrated CRITIC can be developed and applied to other sectors; and finally, the stability of the suggested study against rank-reversal events may be assessed by other academics using the suitable analytic techniques.

The limitations of the study can be summarized as follows: (i) Within the automotive industry, there are several manufacturing areas, and the efficacy of various processes in these production areas may be contested. In this study, the automotive industry is examined as a whole; however, a more in-depth examination of its subdomains can be conducted. (ii) This study does not use any pre-existing data sets thus the results exclusively rely on subjective evaluations. An MCDM model can be developed that uses actual data by using machine learning methods e.g., logistic regression, linear discriminant analysis, and decision trees. (iii) Pythagorean fuzzy sets allow users model the membership and non-membership parameters, and the hesitancy parameter is directly derived from these values. However, in certain circumstances user may prefer to set hesitancy parameters independently. (iv) The CRITIC approach’s inherent characteristics require the calculation of correlation coefficients. Due to the inability to do this computation in a Pythagorean fuzzy environment, the the problem is transferred to a crisp environment, which may cause information loss.

## Supporting information

S1 Data (XLSX)

S1 Table (DOCX)
