# Supplementary material for: Additive manufacturing process selection for automotive industry using Pythagorean fuzzy CRITIC EDAS
Source: PLoS One. 2023 Mar 9;18(3):e0282676. doi: 10.1371/journal.pone.0282676 (PMC9997986; doi:10.1371/journal.pone.0282676)
Supplement: S1 Table — (DOCX) [file pone.0282676.s002.docx]

Table 1. Evaluation of AM Processes with MCDM Methods in the Literature

| **Authors** | **MCDM Methods** | **Processes** | **Evaluation Criteria** |
| --- | --- | --- | --- |
| Shende and Kulkarni (2014) | GT&MA, TOPSIS | SLA, SLS, FDM, 3DP | Accuracy, surface finish, tensile strength, elongation, heat deflection temperature, part cost, build time |
| Vinodh et al. (2014) | Fuzzy VIKOR | SLA, SLS, FDM | Surface quality, building time, support structure, build speed, build material, surface finish, maximum part size, variet, production cost, price, complexity, reliability, flexibility, lead time improvement potential, cost improvement potential, office friendliness, waste disposal, recycling, employee training, skill required, local availability of technology |
| Mancanares et al. (2015) | AHP | SLA, 3DP, SLS, FDM, DMLS, CJP, MJP | Technology, materials, size, multicolor feature, resolution, layer thickness, accuracy, speed, power specs, weight, price |
| Vimal et al. (2016) | Fuzzy ANP, Fuzzy TOPSIS | SLA, SLS, LENS, 3DP | Mechanical properties, process capability, manufacturing efficiency, cost, footprint, process emission, resource utilization |
| Anand et al. (2018) | Fuzzy AHP, Fuzzy TOPSIS | LIFT, micro-SLA, micro-SLS, Deposited nanoparticle inkjet, micro-3DP | Layer thickness, resolution, roughness, minimum feature size, speed, aspect ratio, strength, material compatibility, cost, geometric complexity |
| Peko et al. (2018) | AHP, Fuzzy AHP, PROMETHEE | 3DP, FDM, SLS, Photopolymer Jetting | Dimensional accuracy, surface roughness, mechanical properties, process cost, process time, post-processing |
| Zaman et al. (2018) | AHP, SAW | MJM, SAS, DLP, FDM | Material strength properties, surface finish, geometry complexity, accuracy, layer thickness, build volume, material cost, material usage efficiency, machine cost, labor cost, build speed |
| Khamhong et al. (2019) | AHP | Only a framework for prioritizing assessment criteria for use in evaluating AM processes is presented. | Accuracy, finish surface, part cost, build time, part smoothness, tensile strength, elongation, cost, printer cost, max build size, user preference |
| Liu et al. (2020) | AHP | FDM, SLM, SLS, MJP, EBM, HSS | Accuracy, surface quality, tensile strength, yield strength, flexural strength, elongation at break, build volume |
| Raigar et al. (2020) | BWM, PIV | VAT, ME, PBF, MJ | Dimensional accuracy, surface roughness, tensile strength, percentage elongation, heat deflection temperature, process cost, build time |
| Bikas et al. (2021) | AHP | VAT, MJ, BJ, LPBF, EBM, DED, SL, extrusion | Overhangs, bridging, bores and channels, thickness, size, and surface roughness criteria |
| Ren et al. (2022) | CPC, BWM | ME, VAT, BJ, MJ, DED, PBF | Cost, printing speed, surface finish, autoignition temperature, Young’s modulus, dimensional accuracy, melting point |
